# The Story of the Insane from Year to Year

**Published:** 1906-08-25

**Authors:** 


### THE STORY OF THE INSANE FROM YEAR TO YEAR.

Eighteenth Annual Series.

### THE RETREAT, YORK.

At this asylum the number of patients was almost the same at the beginning and end of the year, being 166 at the former, and 167 at the latter. There were 43 admissions; 13 recoveries; 10 discharged relieved; 9 discharged not improved, and 10 died. The total number under treatment was 209, and the average daily number resident was 168. The recoveries were 31.70 per cent. of the admissions, and the deaths were 5.92 per cent. of the average number resident. Of the deaths 5 were caused by cerebral diseases, and 1 by pleuro-pneumonia. There was an outbreak of scarlet fever during the year, and Dr. Pierce was one of the sufferers. There was no fatal case, but only those who have lived in an asylum can fully realise what an epidemic of this kind means for the staff, especially when it appears, as in this instance, during Christmas, when everyone is fully occupied. As might have been expected there was a falling-off in the income of the Institution; but we are glad to learn that there is no intention on the part of the Management Committee either to enlarge the asylum or to reduce the assistance given to the poorer patients. A legacy of £1,000 was received from a relative of one of the patients, and it is to be desired that such marks of appreciation were oftener found in asylums, and that donations were oftener given to those lunatic hospitals which, life the Retreat, may still be considered as in great part "Charities." When speaking of the recoveries, Dr. Pierce points out the fact that patients often recover in asylums when private treatment, even under what seems favourable conditions, has failed; and he thinks this may be owing to the greater attention a patient receives in an asylum than he can in a private house, and, he adds, that plain, candid speaking is a simple matter in an asylum, but by no means so in private practice. There is an admirable system of training nurses in this asylum. The course lasts four years, and nurses joining the staff must sign an agreement to take the four years' course. This rule will tend to prevent that roving about asylums which is too common among the nurses, and, as Dr. Pierce puts it, will "discourage applicants who do not seriously intend following the profession of nursing, and indirectly this raises the social status of the nursing staff." In all, nineteen nurses have gone through the four years' course, and were awarded the William Tuke Medal. The rates of admission for patients vary from £2 8s. to £7 7s. a week; but the charitable assistance given to those who cannot afford even the lowest of these sums is very great. More than 50 per cent. pay less than their cost, and 21 per cent, pay only 12s. a week.

